# The Menopause Rating Scale (MRS) scale: A methodological review

**DOI:** 10.1186/1477-7525-2-45

**Published:** 2004-09-02

**Authors:** Klaas Heinemann, Alexander Ruebig, Peter Potthoff, Hermann PG Schneider, Frank Strelow, Lothar AJ Heinemann, Do Minh Thai

**Affiliations:** 1 Center for Epidemiology & Health Research Berlin, Invalidenstr. 115, 10115 Berlin, Germany; 2 Berlex Canada Inc., 334 Avenue Avro, Pointe-Claire (Québec) H9R 5W5, Canada; 3 TNS Healthcare(Germany), Landsberger Str. 338, D-80687 Munich, Germany; 4 University Muenster, Dept. Obstetrics and Gynecology, Von-Esmarch-Strasse 56, 48149 Muenster, Germany; 5 Schering Deutschland GmbH, Max-Dorn-Str. 10, 10589 Berlin, Germany

**Keywords:** MRS, Health Related Quality of Life, Questionnaires, Reliability, Validity

## Abstract

**Background:**

This paper compiles data from different sources to get a first comprehensive picture of psychometric and other methodological characteristics of the Menopause Rating Scale (MRS) scale. The scale was designed and standardized as a self-administered scale to (a) to assess symptoms/complaints of aging women under different conditions, (b) to evaluate the severity of symptoms over time, and (c) to measure changes pre- and postmenopause replacement therapy. The scale became widespread used (available in 10 languages).

**Method:**

A large multinational survey (9 countries in 4 continents) from 2001/ 2002 is the basis for in depth analyses on reliability and validity of the MRS. Additional small convenience samples were used to get first impressions about test-retest reliability. The data were centrally analyzed. Data from a postmarketing HRT study were used to estimate discriminative validity.

**Results:**

*Reliability *measures (consistency and test-retest stability) were found to be good across countries, although the sample size for test-retest reliability was small.

*Validity: *The internal structure of the MRS across countries was astonishingly similar to conclude that the scale really measures the same phenomenon in symptomatic women. The sub-scores and total score correlations were high (0.7–0.9) but lower among the sub-scales (0.5–0.7). This however suggests that the subscales are not fully independent.

Norm values from different populations were presented showing that a direct comparison between Europe and North America is possible, but caution recommended with comparisons of data from Latin America and Indonesia. But this will not affect intra-individual comparisons within clinical trials.

The comparison with the Kupperman Index showed sufficiently good correlations, illustrating an adept criterion-oriented validity. The same is true for the comparison with the generic quality-of-life scale SF-36 where also a sufficiently close association has been shown.

**Conclusion:**

The currently available methodological evidence points towards a high quality of the MRS scale to measure and to compare HRQoL of aging women in different regions and over time, it suggests a high reliability and high validity as far as the process of construct validation could be completed yet.

## Background

The interest of clinical research in aging women and males increased in recent years and thereby the interest to measure health-related quality of life and symptoms. Women, as do men, experience an age-related decline of physical and mental capacity. They observe symptoms such as periodic sweating or hot flushes, impaired memory, lack of concentration, nervousness, depression, insomnia, and bone – joint complaints.

The Menopause Rating Scale (MRS) is a health-related quality of life scale (HRQoL) and was developed in response to the lack of standardized scales to measure the severity of aging-symptoms and their impact on the HRQoL in the early 1990s. Actually, the first version of the MRS was to be filled out by the treating physician but methodological critics lead to a new scale which can easily be completed by *women, *not by their physician [1,2].

The validation of the MRS started some years ago [2-6] aiming at establishing an instrument to measure HRQoL that can easily be completed. The aims of the MRS were (1) to enable comparisons of the symptoms of aging between groups of women under different conditions, (2) to compare severity of symptoms over time, and (3) to measure changes pre- and post-treatment [4-6]. The MRS was formally standardized according to psychometric rules and initially published in German [2]. During the standardization of this instrument, three independent dimensions were identified explaining 59% of the total variance (factor analysis): psychological, somato-vegetative, and urogenital sub-scale. The MRS consists of a list of 11 items (symptoms or complaints). Each of the eleven symptoms contained in the scale can get 0 (no complaints) or up to 4 scoring points (severe symptoms) depending on the severity of the complaints perceived by the women completing the scale (an appropriate box is to be ticked).

The scoring scheme is simple, i.e. the score increases point by point with increasing severity of subjectively perceived symptoms in each of the 11 items (severity 0 [no complaints]...4 scoring points [very severe symptoms]). The respondent provides her personal perception by checking one of 5 possible boxes of "severity" for each of the items. This can be seen in the questionnaires in the additional files linked to this publication. The composite scores for each of the dimensions (sub-scales) is based on adding up the scores of each item of the respective dimensions. The composite score (total score) is the sum of the dimension scores. The three dimensions, their corresponding questions and the evaluation are detailed and summarized in an attached file linked to this publication [see Additional file 1].

The MRS scale became internationally well accepted. The first translation was into English [7]. Other translations followed [8], i.e. taking international methodological recommendations [9,10] into consideration. Currently, the following versions are available: Brazilian, English, French, German, Indonesian, Italian, Mexican/Argentine, Spanish, Swedish, and Turkish language. These versions are available in a published form, and can be downloaded in PDF-format from the internet (see reference 8 and ).

Like in other QoL scales, it is a challenge to satisfy the demands of a clinical utility and outcomes sensitivity, and this in addition to the conventional psychometric requirements of test reliability and validity.

The aim of this paper is to present additional psychometric data to discuss the methodologically relevant characteristics of the MRS scale.

## Methods

The development of the scale, instrument characteristics (item selection, scaling), and norms and standardized scores have been published elsewhere [2-5]. This applies also for a few data that have been published on test-retest stability and criterion-dependent validity [3,6].

During the last two years a number of smaller and larger investigations were made from different groups to further check methodological features of the scale. We performed recently a large, multinational survey to represent the situation across nine countries and cultures using existing and for the respective countries representative panels between November 2001 and February 2002 to get information about knowledge, attitudes and behaviour related to hormonal treatment in women aged 40–70 years: *Europe *(Germany, France, Spain, Sweden), *North America *(USA), *Latin America *(Mexico, Argentine, Brazil), and as example for *Asia *– Indonesia. Study participants were accrued as a random sample of females aged 40 to 70 years from existing population panels. The sample size in each of the countries was about 1000 females aged 40–70 years, with exception of USA (n = 1500). The participation rates ranged between 46 and 94% across countries. The demographic details of the sample are: On average, about tertiles of the respondents were under 50 years, between 50–59, and over 60 years old in most of the countries, however, about 50% were less than 50 years in Indonesia and in Brazil. The majority of respondents reported a Christian religion in Europe (range: 74% (Germany) to 96% (Spain), 85% in USA, and in Latin America (range: 95% (Argentina) to 97% (Mexico). The use of the MRS was part of this survey, i.e. multinational data became available to reconsider methodological issues more thoroughly such as internal structure of the scale, reliability (internal consistency alpha), and reference values for different population.

For the purposes of reliability assessment we performed a few preliminary studies with a test-retest approach. These small, descriptive studies of community samples of women aged 40–70 were done in summer and fall 2002 by local collaborators in the respective countries, but they were done separately and independent from the main study. These studies were done just for orientation with convenience samples – not representative for the respective population.

There is only one intervention study (before and after hormonal treatment) available to our knowledge. This study has been published [6] but not with regard to methodologically relevant results of the MRS. These data will be published soon.

With these data available, we were able to scrutinize many methodological characteristics of the MRS scale to review most fundamental psychometric characteristics as well validity parameters.

## Results and Discussion

### Reliability

The assessment of scientific measurements depends first of all on the evidence of replicability (consistency) and test-retest reliability. In contrast to systematic and random variation, reliability gives an estimate of method-related measurement error which should be low not to hide or dilute intended systematic changes – due to treatment for example.

Table 1 show the internal consistency measured with Cronbach's Alpha. The consistency coefficients range between 0.6 and 0.9 across countries for the total score as well the scores in the three domains. This is indicative for a very acceptable consistency of the MRS scale in our opinion. Moreover, there is no evidence that the scale works different in so many different countries in four continents.

**Table 1:** Internal consistency coefficients (alpha) for the MRS scale across countries: total score and scores for the psychological, somatic, and urogenital domain. Data from the Nine-Country Study

	**International**	**Europe**					**North America**	**Latin America**			**Asia**	
	**Total**	**Overall**	Sweden	Germany	France	Spain	**USA**	**Overall**	Mexico	Brazil	Argentina	**Indonesia**

N	**9907**	**4465**	1490	1050	941	984	**1440**	**3002**	1002	1000	1000	**1000**
Total score	**0.83**	**0.86**	0.85	0.84	0.86	0.86	**0.88**	**0.86**	0.87	0.86	0.83	**0.84**
Psychologic ascore	**0.87**	**0.88**	0.88	0.86	0.89	0.86	**0.90**	**0.85**	0.86	0.87	0.81	**0.79**
Somatic score	**0.66**	**0.64**	0.65	0.64	0.64	0.61	**0.70**	**0.66**	0.65	0.69	0.64	**0.69**
Urogenital score	**0.65**	**0.65**	0.64	0.63	0.67	0.67	**0.70**	**0.60**	0.62	0.55	0.62	**0.65**

The test-retest correlation coefficients (Pearson's correlation) support the suggestion of a good temporal stability of the total scale and its three sub-scales (Table 2), although most of the assessments across countries are based on very small numbers and convenience samples not claiming to be representative for the respective population. The intention of these pilot studies was to get a preliminary idea about retest stability. Larger sample sizes are required to permit final conclusions for individual countries / languages.

**Table 2:** Test-retest coefficients (Pearson's correlation coefficient "r") for the MRS scale across countries: total score and scores for the psychological, somatic, and sexual sub-scale.

	All	Europe								Latin America			Asia	
	
	Overall	**Overall**	Germany	UK	France	Spain	Portugal	Sweden	Turkey	**Overall**	Argentina	Brazil	**Overall**	Indonesia
N	349	**259**	73	30	36	30	30	30	30	**60**	30	30	**30**	30
Total score	0,90	**0,92**	0,82	0,80	0,89	0,93	0,96	0,90	0,90	**0,81**	0,78	0,82	**0,84**	0,84
Psychological score	0,84	**0,87**	0,76	0,72	0,88	0,92	0,91	0,79	0,82	**0,72**	0,66	0,76	**0,71**	0,71
Somatic score	0,89	**0,90**	0,80	0,85	0,82	0,88	0,97	0,95	0,93	**0,85**	0,86	0,85	**0,81**	0,81
Urogen. score	0,86	**0,89**	0,77	0,82	0,94	0,98	0,95	0,87	0,89	**0,73**	0,67	0,74	**0,50**	0,50

The test-retest coefficients of the total score range between 0.8 and 0.96 across Europe, North and Latin America, and Asia. When it comes to the subscales with much fewer items, the variation increased and some of the coefficients went down to 0.5 (urogenital domain in Indonesia). Altogether, the test-retest stability over a time period of two weeks aggregated at the international level supports the notion of a very acceptable test-retest reliability of the total scale and their three sub-scales.

Although there is an impressive set of information currently available concerning the reliability of the MRS scale, there are also limitations: Small sample sizes prevent a final conclusion regarding test-retest reliability in some of the languages the scale has been translated in.

### Validity

Similar to reliability which assesses the consistency of measurement, the validity estimates if a scale measures what it intends to measure. But whereas reliability can be determined straight forward with very few indicators, the validity is almost always a continuous process (construct validation). It is a process of accumulating evidence for a valid measurement of what is purposed. Therefore, the currently available data are already fairly comprehensive and do pave the way for a focussed and continuing validation process.

### Internal structure of the MRS across countries

The first step of validation is usually to multivariately demonstrate a similar internal structure ("dimensions") of a given scale through factor analysis.

The first factorial analysis in 1996 was applied to identify the dimensions of the scale. Three dimensions of symptoms/complaints were identified [2]: a psychological, a somato-vegetative, and a urogenital factor that explained 58.8% of the total variance.

The recent large, multinational survey in nine countries of four continents provided data to compare with the initial standardisation sample of the MRS. The question was: Is the internal structure of the MRS results comparable among different countries or cultures. Astonishingly similar factor loadings of the 11 items of the 3 domains of the MRS were observed (Table 3). The same applies for the individual countries of the respective regions (data not shown). Although the prevalence of menopausal symptoms may slightly differ among regions/cultures (see later), the structure of complaints/symptoms seems to be pretty much the same. It suggests that the scale measures constantly the same phenomenon which speaks in favour of the translation/cultural adaptation of the scale.

**Table 3:** Internal structure of the MRS scale across countries in 9 countries of four continents (2002) compared with the initial analysis of a German sample in 1996. Principal component analysis, Varimax rotation. Complaints (item number in MRS), numbers, and country groups. Only factor loadings ≥ 0.5 are shown.

		Complaints (item number)										
	N	Flushes(1)	Heart(2)	Sleep(3)	Joints(11)	Depress(4)	Irritabil(5)	Anxiety(6)	Exhaust(7)	Sexual(8)	Bladder(9)	Dryness(10)
**Germany, 1996**	479											
somatic		0.8	0.7	0.6	0.5							
psychologic						0.8	0.7	0.8	0.6			
urogenital										0.7	0.8	0.8
**All countries, 2002**	10297											
somatic		0.7	0.8	0.5	0.5							
psychologic						0.8	0.8	0.8	0.7			
urogenital										0.7	0.6	0.8
**Europe, 2002**	4791											
somatic		0.7	0.7	0.6	0.6							
psychologic						0.8	0.9	0.8	0.6			
urogenital										0.7	0.6	0.8
**N.-America (USA)**	1500											
somatic		0.7	0.7	0.7	0.5							
psychologic						0.8	0.9	0.8	0.6			
urogenital					0.5					0.8	0.6	0.8
*Latin America, 2002*	3006											
somatic		0.5	0.9	0.5	0.4							
psychologic				0.5		0.8	0.8	0.8	0.7			
urogenital										0.6	0.7	0.8
**Asia (Indonesia)**	1000											
somatic		0.9	0.6	0.5	0.5							
psychologic						0.8	0.8	0.8	0.5			
urogenital										0.8	0.5	0.9

However, there are indications that the domains could be somewhat overlapping and not as entirely independent as the statistical model suggests: Muscle or joint problems got a loading of 0.5 in the somatic and urogenital domain (USA), and sleep disturbances both 0.5 in somatic and psychological domain (Latin America). These two items had similar problems in Spain, Mexico, and Brazil but not in other countries (data not presented in table 3).

In clinical studies intra-individual comparisons over time (before/after treatment) will be the main criterion which might not be affected by potential slight differences in the patient reported outcome structure. Therefore the general agreement in the internal structure of the MRS scale across country groups, even accepting the possibility of slight differences in two items (cf. Table 3), suggests that the scale can very well be used in clinical studies – even including different countries.

### Sub-scores and total score correlations

The relations among the sub-scales and the aggregate total scale are patterns that are important in the methodological assessment of a scale. In an ideal world, the correlations between subscales (supposed to be independent due to the statistical model) would be closer to 0 than the correlations with the construct of the aggregate total score to which all sub-scales should significantly contribute. But that is theory; Table 4 shows only somewhat lower correlations among sub-scales (0.4–0.7) as compared with correlation of sub-scales with the total score (0.7–0.9). This is less different than one would have wished. It suggests that the sub-scales are not as independent from each other as one would expect them to be – based on a factorial analysis with orthogonal factors. The situation was similar in the four regions listed in Table 4 and in the individual countries belonging to these regions. It is important to realize how similar these correlation coefficients are among countries/aggregates. This is suggestive of pretty similar features of the MRS scale across the countries of this review.

**Table 4:** Domain score – total score correlations of the MRS scale across four country groups. Community samples.

	Domains		
	Psychological score	Somatic score	Urogenital score
**Europe (n = 4246)**			
Total score	0.9	0.9	0.7
Psychological score	--	0.6	0.5
Somatic score	--	--	0.5
**North America (n = 1376)**			
Total score	0.9	0.9	0.8
Psychological score	--	0.7	0.5
Somatic score	--	--	0.5
**Latin America (n = 3001)**			
Total score	0.9	0.9	0.7
Psychological score	--	0.7	0.5
Somatic score	--	--	0.5
**Asia (n = 1000)**			
Total score	0.9	0.9	0.7
Psychological score	--	0.7	0.4
Somatic score	--	--	0.4

### Compatibility of MRS reference values for different population

There are different categories of severity of complaints or problems with QoL. For the comparison of these categories across countries or cultures it is important to understand the prevalence of complaints. Currently, there is only one table with reference values and definitions published – for the German population from the initial standardization of the MRS [2]. Are these reference values applicable for other countries/cultures?

The data of our large multicultural survey permitted such comparisons. The mean values (SD) of the MRS total score and the three domains are depicted in Table 5. The mean values of the total score and the 3 domain scores are not statistically significantly different between Europe and North America. Thus, there is no evidence yet to exclude direct comparisons of MRS values between these regions.

**Table 5:** Mean values and standard deviation of MRS total score and 3 domains. Results from a large, multinational survey (see methods)

	Total score		Psychological Score		Somato-vegetative Score		Urogenital Score	
	n	Mean (SD)	n	Mean (SD)	n	Mean (SD)	n	Mean (SD)
Europe	4246	8.8 (7.1)	4453	3.4 (3.4)	4465	3.6 (2.9)	4465	1.9 (2.2)
N.-America (USA)	1376	9.1 (7.6)	1426	3.4 (3.5)	1440	3.8 (3.1)	1437	2.0 (2.3)
Lat.-America	3001	10.4 (8.8)	3002	4.9 (4.5)	3006	4.1 (3.6)	3005	1.4 (2.2)
Asia	1000	7.2 (6.0)	1000	2.9 (2.9)	1000	3.3 (2.7)	1000	1.0 (1.6)

However, the total, psychological and somatic scores were systematically higher in Latin America, and systematically (significantly) lower in Asia (Indonesia) than in Europe/North America. The urogenital scores were significantly lower in Latin America/Indonesia than in Europe/US. Obviously the perception of the prevalent symptoms depends on cultural factors – or the symptoms show real differences in prevalence. Thus, direct comparisons of MRS scores between Europe/North America on the one side and regions in Latin America and Asia cannot are not recommended. This does not effect intra-individual comparisons (e.g., pre/ post therapy) within these countries and it may also very little affect the comparison of relative changes (pre/post treatment) among different countries. The latter is a hypothesis and needs further evidence form research/experience.

Similar mean values could still have a different distribution across the proposed four categories of "severity of complaints": no/little symptoms, mild, moderate, and severe complaints, i.e. for the total scale and the three domains. The prevalence of these categories across the four regions studied is seen in Table 6. The comparison of the prevalence (and 95% confidence interval) showed that the above discussed differences between Europe/US and Latin-America or Indonesia very much depend on the severity of complaints. Whereas the differences in the psychological domain were less impressive, the dissimilarity was most pronounced in the urogenital domain and less also in the somatic domain. Whether this is due to different perception of identical symptoms (differences in the appearance of symptoms or both) remains a speculation. This however needs to be considered when direct comparisons among different cultures are intended. The prevalence of different "degrees of severity" of menopausal symptoms measured with the MRS was found to be almost identical in the aggregate of Europe and North America.

**Table 6:** Comparison of "degree of severity" of the MRS and its domains. Prevalence in percent (%) and 95% confidence interval (in parenthesis) in the population sample studied in the respective regions (see methods)

	**Europe**	**North America**	**Latin America**	**Asia**
**Total score**				
No, little (0–4)	28.8 (+/-1.3)	28.0 (+/-2.3)	31.0 (+/-1.7)	40.2 (+/-3.0)
Mild (5–8)	21.9 (+/- 1.2)	23.9 (+/-2.2)	20.2 (+/-1.4)	27.5 (+/-2.8)
Moderate (9–16)	25.1 (+/-1.2)	25.7 (+/-2.2)	26.2 (+/-1.6)	22.8 (+/-2.6)
Severe (17+)	24.3 (+/- 1.2)	22.5 (+/-2.1)	22.7 (+/-1.5)	9.5 (+/-1.8)
**Psychological domain**				
No, little (0–1)	35.4 (+/-1.4)	36.8 (+/-2.4)	36.8 (+/-1.6)	41.3 (+/-3.1)
Mild (2–3)	21.8 (+/-1.2)	21.9 (+/-2.1)	21.9 (+/-1.4)	25.4 (+/-2.7)
Moderate (4–6)	19.5 (+/-1.1)	18.7 (+/-2.0)	18.7 (+/-1.4)	21.3 (+/-2.6)
Severe (7+)	23.4 (+/-1.2)	22.5 (+/-2.1)	22.5 (+/-1.7)	12.0 (+/-2.0)
**Somato-vegetative domain**				
No, little (0–2)	39.5 (+/-1.4)	37.9 (+/-2.4)	42.1 (+/-1.8)	46.8 (+/-3.1)
Mild (3–4)	22.6 (+/-1.2)	25.6 (+/-2.2)	19.4 (+/-1.4)	27.0 (+/-2.8)
Moderate (5–8)	24.2 (+/-1.2)	24.3 (+/-2.2)	25.6 (+/-1.6)	20.8 (+/-2.5)
Severe (9+)	13.7 (+/-1.0)	12.1 (+/-1.7)	12.9 (+/-1.2)	5.4 (+/-1.4)
**Urogenital domain**				
No, little (0)	34.3 (+/-1.3)	33.4 (+/-2.4)	28.2 (+/-1.8)	55.6 (+/-3.1)
Mild (1)	17.2 (+/-1.1)	17.0 (+/-2.0)	18.6 (+/-1.1)	18.6 (+/-2.4)
Moderate (2–3)	23.0 (+/- 1.2)	24.2 (+/-2.2)	21.8 (+/-1.3)	17.0 (+/-2.3)
Severe (4+)	25.6 (+/-1.2)	25.4 (+/-2.2)	31.4 (+/-1.3)	8.8 (+/-1.8)

### Criterion-oriented validity: correlation with other scales

In fact, the comparison with other scales of similar purpose is important. It is known from other quality of life scales that comparisons with scales with similar purposes are much more important than comparisons with so-called objective parameters such as exercise tests, physiological or chemical parameters – in our case with hormones.

Health related quality of life should be validated against quality of life measured with other generic QoL scales (e.g., SF-36), and against specific instruments to measure symptoms in aging women (e.g. Kupperman index). These data were published elsewhere [6,11] but will be briefly summarized in the context of this review.

### Kupperman Index

Although the Kupperman Index was not validated according to psychometric standards it is still in use in the medical practice to monitor menopausal symptoms. Therefore a comparison with the fully standardized MRS seems to be reasonable. If one divides the distribution of both scales into quartiles and compares the frequencies, both instruments were found to be closely associated: Kendall's tau-b coefficient 0.75 (95% CI 0.71–0.80) [6]. Similar was the Pearson correlation coefficient with r= 0.91(95% CI 0.89–0.93). The two scales can be regarded as measuring the same phenomena. However, some methodological problems of the Kupperman Index were identified in this comparison (see [6] for details).

### Generic QoL Scale SF-36

Two sub-scales of the multi-domain quality of life scale SF-36 was compared with the MRS: the somatic sum score (with somatic domain of MRS) and the psychologic sub-scales of both instruments. Both somatic domains were sufficiently well and significantly associated: Kendall's tau-b = 0.43 (95% CI 0.52–0.35); Pearson correlation coefficient r= 0.48 (95% CI 0.58–0.37). That means, the higher the score in the somatic dimension of the MRS, the lower the quality of life according to the somatic sum-score of the SF-36 [6,11]. Similar was the results of the comparison of the psychological scores of both instruments: Kendall's tau-b = 0.49 (95% CI 0.56–0.41); Pearson correlation coefficient r= 0.73 (95% CI 0.81–0.65).

#### Discriminative validity

i.e., the ability of the scale to accurately measure treatment effects and to predict the clinically based assessment of physicians, was not analysed so far. At present, there is one post-marketing study that can be used to preliminary assess discriminative validity. The results will be published soon elsewhere. To this end, many clinicians understand the term "validity" and mean high utility for clinical work or research.

## Conclusions

The MRS scale is a standardized HRQoL scale with good psychometric characteristics. The use in many countries offered the possibility to compare the test characteristics across countries. Reliability measures (consistency and test-retest stability) were found to be good in all countries where data were obtained – however, some samples were very small and therefore considered as preliminary information.

The validity was measured in its various forms: The internal structure of the MRS across countries was sufficiently similar to conclude that the scale really measures the same phenomenon in women with complaints. The sub-sores and total score correlations showed high coefficients with the total score and less among the sub-scales. This however indicates that the subscales are not fully independent in practice.

Comparisons of reference values from different populations showed that the MRS scores can easily be compared between Europe and North America/US. Direct comparisons between Europe/North America and Latin American countries and Asia (Indonesia) should be considered with caution because the severity of reported symptoms seems to differ. The reasons are not clear, further research is needed.

The comparison with other scales for menopausal symptoms (Kupperman Index) showed a sufficiently close association and correlation coefficients, i.e. illustrating a good criterion-oriented validity. The same is true for the comparison with the generic QoL scale SF-36 where also high correlation coefficients have been shown.

Thus, the currently available methodological evidence points towards a high quality of the MRS scale to measure and to compare HRQoL of aging males over time or intervention. It suggests a high reliability and high validity as far as the process of construct validation could be completed.

## Authors' contributions

KH: responsible for drafting the manuscript and running analyses. AR: responsible for designing and overseeing the multinational survey (2001/2002), contributed to writing and revising of the paper. PP: co-ordination of the field work of the multinational survey, setting up the initial database, and contributed to writing of the paper, responsibility in developing/validating the MRS scale. HPGS: Major responsibility in developing the MRS scale, contributed to writing/revision of the manuscript. FS: Provided data of a clinical study, contributed to writing of the manuscript. LAJH: responsible for the collection and evaluation of the data, and involved in writing/revising the paper. DMT: responsible for checking the integrated database, responsible for several analyses regarding validity, and contributed to writing of the paper.
